# Spatial, temporal, and spatiotemporal cluster detection of malaria incidence in Southwest Ethiopia

**DOI:** 10.3389/fpubh.2024.1466610

**Published:** 2025-01-13

**Authors:** Lidetu Demoze, Fetlework Gubena, Eyob Akalewold, Helen Brhan, Tigist Kifle, Gelila Yitageasu

**Affiliations:** ^1^Department of Environmental and Occupational Health and Safety, Institute of Public Health, College of Medicine and Health Sciences, University of Gondar, Gondar, Ethiopia; ^2^Department of Epidemiology and Biostatistics, Institute of Public Health, College of Medicine and Health Sciences, University of Gondar, Gondar, Ethiopia

**Keywords:** malaria, incidence, spatial, temporal, spatiotemporal, cluster, South Omo Zone

## Abstract

**Background:**

Malaria is a major global health hazard, particularly in developing countries such as Ethiopia, where it contributes to high morbidity and mortality rates. According to reports from the South Omo Zone Health Bureau, despite various interventions such as insecticide-treated bed nets and indoor residual spraying, the incidence of malaria has increased in recent years. Therefore, this study aimed to assess the spatial, temporal, and spatiotemporal variation in malaria incidence in the South Omo Zone, Southwest Ethiopia.

**Methods:**

A retrospective study was conducted using 4 years of malaria data from the South Omo Zone District Health Information Software (DHIS). The incidence rate of malaria per 1,000 people was calculated using Microsoft Excel software. Kulldorff SaTScan software with a discrete Poisson model was used to identify statistically significant spatial, temporal, and spatiotemporal malaria clusters. Graduated color maps depicting the incidence of malaria were generated using ArcGIS 10.7 software.

**Results:**

Spatial clusters were identified in the districts of Dasenech (RR = 2.06, *p* < 0.0001), Hamer (RR = 1.90, *p* < 0.0001), Salamago (RR = 2.00, *p* < 0.0001), Bena Tsemay (RR = 1.71, *p* < 0.0001), Malie (RR = 1.50, *p* < 0.0001), Nyngatom (RR = 1.91, *p* < 0.0001) and North Ari (RR = 1.05, *p* < 0.0001) during the period from 08th July 2019 to 07th July 2023. A temporal cluster was identified as the risk period across all districts between 08th July 2022 and 07th July 2023 (RR = 1.59, *p* = 0.001). Spatiotemporal clusters were detected in Dasenech (RR = 2.26, *p* < 0.001) Salamago, (RR = 2.97, *p* < 0.001) Hamer (RR = 1.95, *p* < 0.001), Malie (RR = 2.03, *p* < 0.001), Bena Tsemay (RR = 1.80, *p* < 0.001), Nyngatom (RR = 2.65, *p* < 0.001), North Ari (RR = 1.50, *p* < 0.001), and Jinka town (RR = 1.19, *p* < 0.001).

**Conclusion:**

Significant spatial, temporal, and spatiotemporal clusters in malaria incidence were identified in the South Omo Zone. To better understand the factors contributing to these high-risk areas, further research is needed to explore individual, household, geographical, and climatic factors. Targeted interventions based on these findings could help reduce malaria incidence and associated risks in the region.

## 1 Introduction

Malaria is an infectious disease prevalent in tropical and subtropical regions worldwide. It is caused by *Plasmodium Protozoa* and transmitted by *Anopheles mosquitoes* (1). The majority of human malaria cases, along with associated morbidity and mortality, are attributed to two *Plasmodium*. Species: *P. falciparum* and *P. vivax* (2). Malaria epidemics have been reported among populations living at altitudes as high as 2,500 meters above sea level (3). Between 2000 and 2015, the number of malaria cases decreased by 27%, while the fatality rate decreased by 60%.

However, since 2015, the drop has halted and even reversed in several nations (4). However, the decrease in malaria occurrence in some regions of the world does not minimize the substantial risk that each person faces when visiting a currently endemic area because travelers from non-endemic areas have not developed immunity to malaria (5). The global tally of malaria cases reached 249 million in 2022, which is up by five million from 2021. The World Health Organization (WHO) African Region accounted for approximately 93.6% of cases and 95.4% of fatalities globally (6). Approximately 35% of the world’s population lives in areas where there is some risk of malaria transmission (7). Among them, approximately 1 billion people reside in regions with a low but still present risk of malaria transmission (7).

In Sub-Saharan Africa, estimates showed a decline in the malaria burden and similar trends, but there was also a resurgence of the malaria burden empirically in some areas (8). In 2020, there were over 200 million malaria cases and 403,000 deaths in Sub-Saharan Africa. Furthermore, in 2022, approximately 249 million malaria cases were reported, with 94% of the cases and 95% of the deaths occurring in the WHO African Region (9). Nigeria (27%), the Democratic Republic of Congo (12%), Uganda (5%), Angola (3.4%), Burkina Faso (3.4%), and Mozambique (4%) accounted for more than half (55%) of all malaria cases worldwide (4). In Ethiopia, 68% of the areas are endemic to malaria, and 60% of the country’s population is prone to infection of malaria (10). In Ethiopia, malaria transmission is seasonal, increasing in September and December following the main rainy season; April and May are months with decreased transmission rates (11). The Ethiopian Ministry of Health reported that 75% of the country, including the Southwest region, is malarious. As a result, approximately 70% of the population is at risk for developing malaria (12–14). Malaria prevention and control services are provided free of charge in the country, and Ethiopia is currently implementing a malaria elimination effort to abolish the disease by 2030 (15, 16).

The implementation of different malaria control initiatives in Ethiopia, such as the use of insecticide-treated bed nets, indoor residual spraying, and treating cases with artemisinin-based combination therapy, resulted in encouraging gains (10, 17). Despite those interventions, malaria contributed to 3% of the total Disability-adjusted life years (DALY) due to all causes in Ethiopia (18). In 2021, Ethiopia constituted 1.7% of the global malaria cases, and there were 2,783,816 cases and 8,041 deaths (19). In addition, malaria cases have soared from the beginning of 2023 to the end of June, and 1,251,910 cases of malaria have been reported in Ethiopia (20). Severe malaria, which causes major organ damage, is more likely in children under the age of five in malaria-endemic nations than in older children and adults, which has a significant impact on local children’s development (21). The South Omo Zone, situated in southwest Ethiopia, is a high-risk area for malaria due to its lowland environment and climatic conditions that support mosquito breeding (22). Malaria incidence in this region, as in other lowland areas of Ethiopia, is heavily influenced by seasonal changes, with transmission rates peaking during the rainy season (23). According to the reports of the South Omo Zone health bureau, despite different interventions such as insecticide-treated bed nets and indoor residual spraying, the incidence of malaria has shown an increment in recent years in the South Omo Zone. Accordingly, this study aims to determine the spatial, temporal, and spatiotemporal variations of malaria incidence in the South Omo Zone, Southwest Ethiopia.

## 2 Materials and methods

### 2.1 Study setting

**Figure 1:**
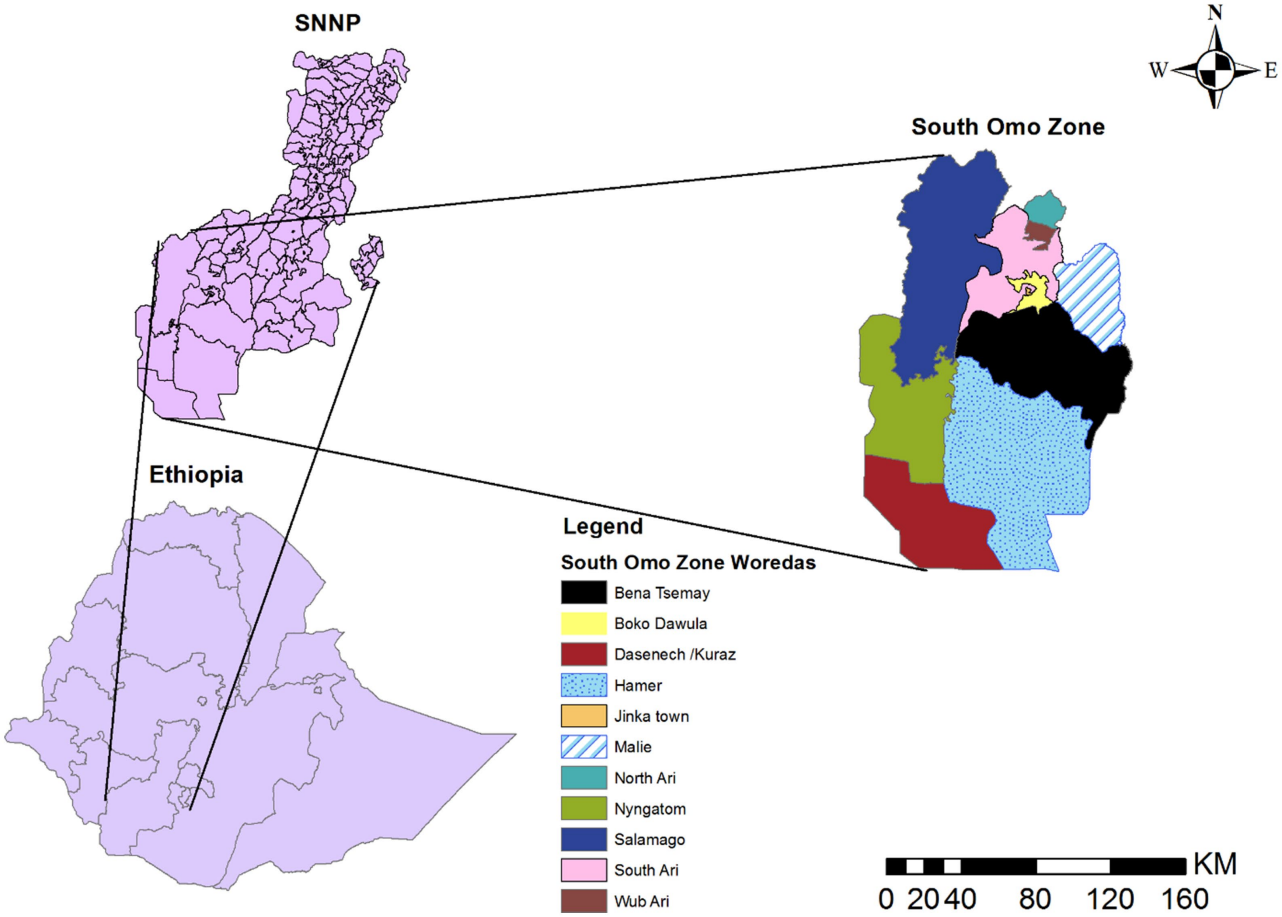
Map of South Omo Zone, Source of shapefile: Ethiopian Statistical Service, own map output from ArcGIS V.10.7.

A retrospective study was conducted from 08th July 2019 to 07th July 2023. The South Omo Zone’s 4 years’ worth of malaria data from the District Health Information Software (DHIS) reporting system was used. The South Omo Zone is located approximately 750 km from Addis Ababa, the capital city of Ethiopia, and 299 km from Hawassa, the capital city of Southern Nations, Nationalities, and Peoples (SNNP) (24). The boundaries of the South Omo Zone are adjoined with the Southwest Ethiopia Peoples Region in the Northwest and North, the Gofa Zone and Basketo Zone in the North, the Gamo Zone in the Northeast, the Oromia Region, Alle Zone, and the Konso Zone in the East, Kenya in the South and South Sudan in the Southwest. The South Omo Zone comprises 10 districts and three administrative towns. These are Salamago, Debub (South) Ari, Semen (North) Ari, Wub Ari, Boko Dawula, Hamar, Bena Tsemay, Dassenech, Malie, and Nyangatom. Currently, there are three administrative towns: Jinka, Gelila, and Turmi. The eight largest ethnic groups are Ari (44.59%), Malie (13.63%), Dasenech (8.17%), Hamer (8.01%), Bena (4.42%), Amhara (4.21%), Tsemai (3.39%), and Nyangatom (2.95%) (25). According to 2023 South Omo Zone plan commission estimates, the total population residing in South Omo Zone is estimated to be 918,440, with 459,586 Malies and 458,854 feMalies in all woredas/districts (woredas, also known as districts, representing the third level of administrative divisions in Ethiopia) (Figure 1).

The South Omo Zone has a large land area and sparsely distributed agro-pastoral communities in the lowlands (while the highland areas are densely populated), and it is overwhelmingly rural (26). The zone is located approximately 400 m above sea level. The average yearly temperature ranges from 18 to 32°C. The average annual rainfall is approximately 390 mm, and rainfall in the study area is irregular and bimodal, falling between September and November and March and May (27). The administrative center of the South Omo Zone is Jinka. The South Omo Zone has four general hospitals and 32 health centers, which serve approximately 1.2 million people in the catchment area.

### 2.2 Data collection

Two nurses and one environmental health professional, all holding Bachelor of Science degrees, extracted data from the DHIS reporting system on a case-by-case basis. They were proficient in DHIS data management and used Microsoft Excel. DHIS refers to the District Health Information Software System, a digital platform used for collecting, managing, and analyzing health data at various administrative levels in Ethiopia. It is used at national, regional, and local levels to track health metrics, including disease surveillance, immunization, maternal and child health, and more. The system integrates data from health facilities nationwide, enabling decision-makers to use this information for planning, monitoring, and evaluation purposes. It supports both communicable and non-communicable diseases and includes tools for data validation, analytics, and reporting. Health facilities in each district treat and record malaria cases based on the World Health Organization (WHO) guideline that defines malaria as the occurrence of malaria infection in a person in whom the presence of malaria parasites in the blood has been confirmed by a Rapid Diagnostic Test (RDT) (28) was collected as confirmed malaria cases. Shapefiles, spatial coordinates (latitudes and longitudes), and population data for each year and each district were obtained from the Ethiopian Statistical Service.

### 2.3 Quality control

The obtained data (confirmed malaria cases) collected by two BScs in nursing and one BSc in environmental health personnel were cross-checked with the district (Woreda and city administration) reporting system. District health offices obtained the missing reports from the zonal health department (DHIS). We cross-verified the DHIS data to ensure the accuracy of the malaria cases entered into the system. The review also aimed to identify any missing reports within the DHIS reporting system. Although the DHIS database is generally reliable, occasional data gaps can occur when some entries fail to reach the zonal health department, where the DHIS is maintained. Our study’s total percentage of missing data in the DHIS was less than 2%. However, we addressed the missing data from the district reporting system. Consequently, our study contains no missing data. The data collectors were informed about the research objectives and data collection procedures. After collection, the data completeness and consistency were checked by the study’s principal investigators before analysis. The final collected data was cleaned, aggregated, edited, checked, and sorted using Microsoft Excel software.

### 2.4 Data management and analysis

Since the study was conducted for district-level malaria incidence cluster detection, the annual population malaria incidence for each district and the average cumulative annual population malaria incidence from 08th July 2019- 07th July 2023 were calculated using Microsoft Excel software and associated with their respective coordinates. The incidence rate is a new case divided by the total population of each district and multiplied by 1,000. Finally, the data was stored in CSV format and exported to ArcGIS version 10.7 (29) and SaTScan™ version 9.6 (30) for further analysis. ArcGIS version 10.7 software was used to analyze spatial autocorrelation (Global Moran’s *I*) and plot the graduated color map. SaTScan™9.6 software was used for purely spatial, temporal, and spatiotemporal analysis. Since the data was counted, the discrete Poisson model was utilized during the spatial, temporal, and spatiotemporal analysis in SaTScan™9.6 software (31).

Spatial autocorrelation analysis (Global Moran’s *I*): A value of −1 indicates that the spatial units are negatively correlated, whereas 1 suggests a positive spatial correlation. If Moran’s I is around 0, there is no spatial correlation (32). A statistically significant Moran’s I with a *p*-value less than 0.05 indicates spatial autocorrelation (32, 33). This analysis was done using ArcGIS version 10.7 software.

Hotspot and Cold Spot Analysis: Identifies statistically significant clusters of high values (hotspots) and low values (cold spots) across the entire study area (34). This analysis was conducted using ArcGIS version 10.7 software.

Purely spatial clusters analysis: A circular window was used to scan the entire study area. The circle’s radius continuously changes from zero to a specified maximum size. The maximum size represents the percentage of the total at-risk population within the scanning window (32). Researchers recommend that the maximum size should not exceed 50%, meaning a reported cluster can include at most 50% of the total population at risk (32, 35, 36). The alternative hypothesis is that the risk within the window varies from the outside risk, while the null hypothesis is that the disease risk is identical inside and outside the scanning window in space (32, 36). The Poisson distribution determines the number of predicted incidences for each circle, which is then compared to the number of observed occurrences inside and outside the window (31, 32, 36). The circle with the maximum Log-Likelihood Ratio (LLR), containing more cases than expected, is identified as the most likely (primary) cluster, indicating it is the least likely to have occurred by chance (36). Based on this, the likelihood ratio within each circle is calculated (36). Under the Poisson assumption, the likelihood function for a specific window is proportional to.


(1) $$\left(\frac{C}{E\left[c\right]}\right){\left(\frac{C-c}{C-E\left[c\right]}\right)}^{C-c}I\left(\;\right)$$

*C* is the total number of malaria incidences, c is the observed number of malaria incidences within the window, and *E[c]* is the expected number of malaria incidences within the window under the null hypothesis (32, 36). Since the analysis will be based on the total number of malaria incidences observed, *C − E[c]* is the expected number of malaria incidences outside the window. *I()* is an indicator function (32). The program will be adjusted to scan for clusters with either high or low rates, then *I() = 1* for all windows (32). The expected number of malaria incidences in each area under the null hypothesis will be calculated using the formula:


(2) $$E\left[c\right]=p*C/P$$

Where c is the observed number of malaria incidences, p is the malaria population in each district, and *C* and *P* are the total numbers of malaria incidences and populations, respectively (32). The significance of the spatial cluster was tested at an alpha threshold of less than 0.05. This analysis was conducted using SaTScan™ 9.6 software.

Purely temporal scan statistics for the temporal cluster: A one-dimensional moving window is only employed when the cylindrical window’s height serves as the time dimension (32). Monte Carlo simulations were used to produce a *p*-value. A significant district was determined by applying a significance criterion of *p* < 0.05. For purely temporal analyses, only the most likely cluster was reported (32). The scan was performed to look for high-rate regions or clusters (32). Analysis was computed using SaTScan™ 9.6 software.

Space–time scan statistics for spatiotemporal clusters: This technique was used to find clusters in time and space (32, 35). It is presumed that the relative risk of malaria incidence within and outside of the window was equal. A cylindrical window with a circular base was used to detect spatiotemporal clusters (32). As in the purely spatial scan statistic, the cylinder’s base represents space, while its height represents time (32, 37). Using a *p*-value found through Monte Carlo simulations, districts with substantial malaria incidence within the corresponding period (time) were found (32). The primary cluster that was least likely to have happened by chance was determined to be the circle with the biggest likelihood ratio and the highest observed incidence of malaria (31, 32). For spatiotemporal analysis, an iterative method outlined by Kulldorff was used to identify secondary clusters for each spatial and space–time scan statistic in addition to the most likely (primary) cluster (32, 36). 50% of the population at risk was designated as the maximum cluster size. Similar to the previous one, a significant district was determined by applying a significance criterion of *p* < 0.05. The analysis was performed using SaTScan™ 9.6 software.

## 3 Results

### 3.1 Distribution of malaria incidence in the South Omo Zone at the district level for each year

**Figure 2:**
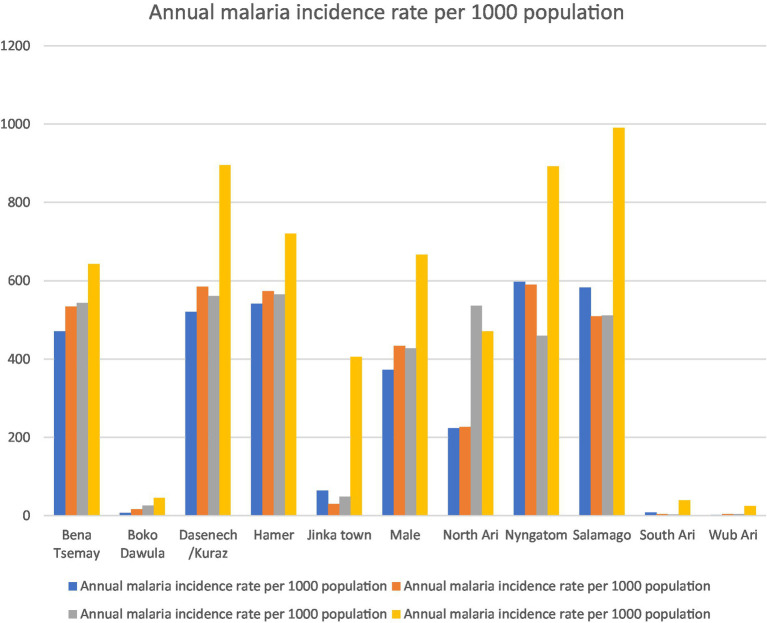
Annual malaria incidence from 2019 to 2023 in districts of the South Omo Zone, Southwest Ethiopia. Source of population data: South Omo Zone plan commission.

The incidence rate showed significant spatial and temporal variation over the study period. Between 2019 and 2023, the highest and lowest incidence rates were 990.4 per 1,000 in Salamago in 2022/2023 and 1.59 per 1,000 in 2019/2020 in the Wub Ari districts, respectively (Figure 2).

### 3.2 Spatial autocorrelation result and graduated color map of malaria incidence

The spatial autocorrelation results indicate that the incidence of malaria was clustered, with a z-score of 2.00 and a *p*-value of 0.045.

**Figure 3:**
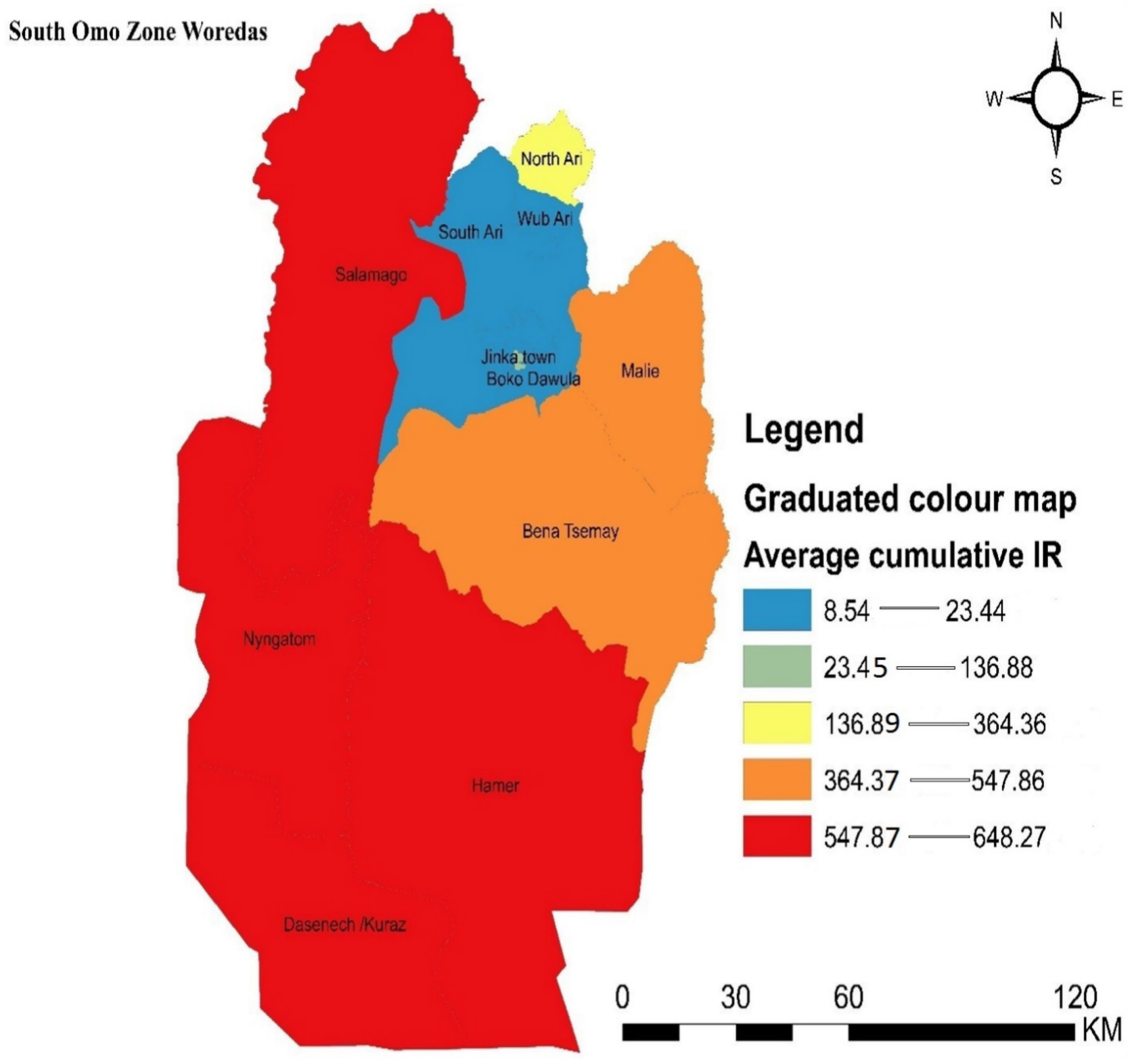
Graduated color map that depicts the spatial distribution of malaria incidence in the South Omo Zone, Southwest Ethiopia, 08th July 2019- 07th July 2023. Source of shapefile: Ethiopian Statistical Service, own map output from ArcGIS V.10.7.

Districts with the highest incidence rates of malaria in the zone were marked in red on the map and were clustered in the southern, southwestern, and northern parts of the study area, including Dasenech, Nyangatom, Hamer, and Saramago. Districts with the second-highest malaria incidence rates were shown in orange and included Bena Tsemay and Malie. Areas with the third-highest malaria incidence rates were shaded in yellow and included North Ari. The districts with the low malaria incidence were located in the northern part of the zone and were indicated by light blue, covering South Ari, Wub Ari, Boko Dawula, and Jinka town (Figure 3).

### 3.3 Hotspot detection

**Figure 4:**
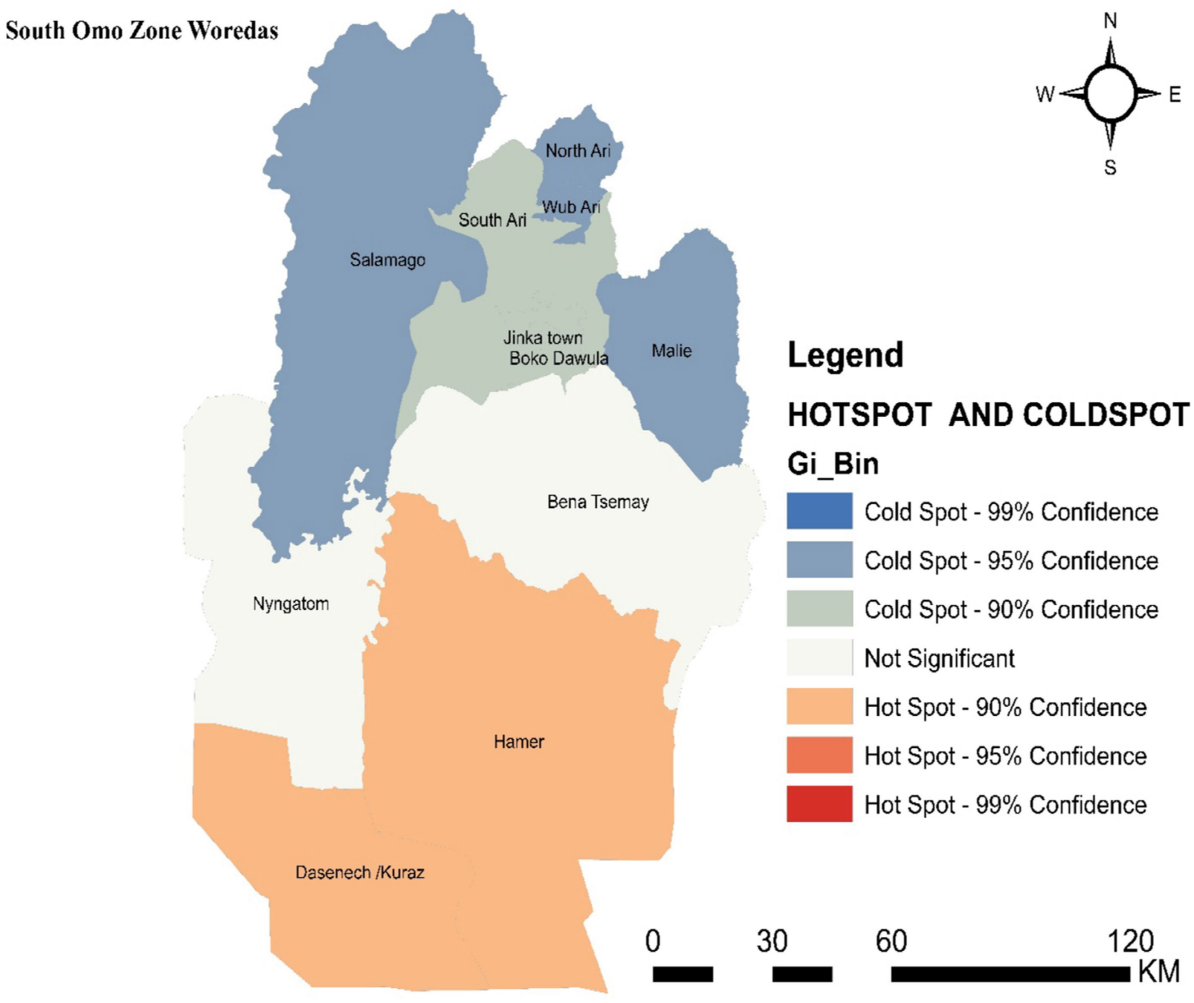
Hot spot detection based on average cumulative annual malaria incidence in the South Omo Zone, Southwest Ethiopia, 08th July 2019- 07th July 2023. Source of shapefile: Ethiopian Statistical Service, own map output from ArcGIS V.10.7.

Hotspot areas with a high cluster of malaria incidence were identified. A hotspot area with high-rate clusters at 90% confidence was observed in Dasenech and Hamer. The hotspots, Dasenech and Hamer, are separated from the cold spots by Nyangatom and Bena Tsemay, which are neither cold nor hot spots. The cold spots include North Ari, Wub Ari, South Ari, Jinka Town, Malie, Salamago, and Boko Dawula. The maximum peak, where spatial clustering was highly pronounced, was at a distance of 62866.1413 meters with a corresponding Z score of 2.001446 (*p*-value <0.05). This distance band was used to analyze hotspot clusters (Figure 4).

### 3.4 Spatial clusters

**Figure 5:**
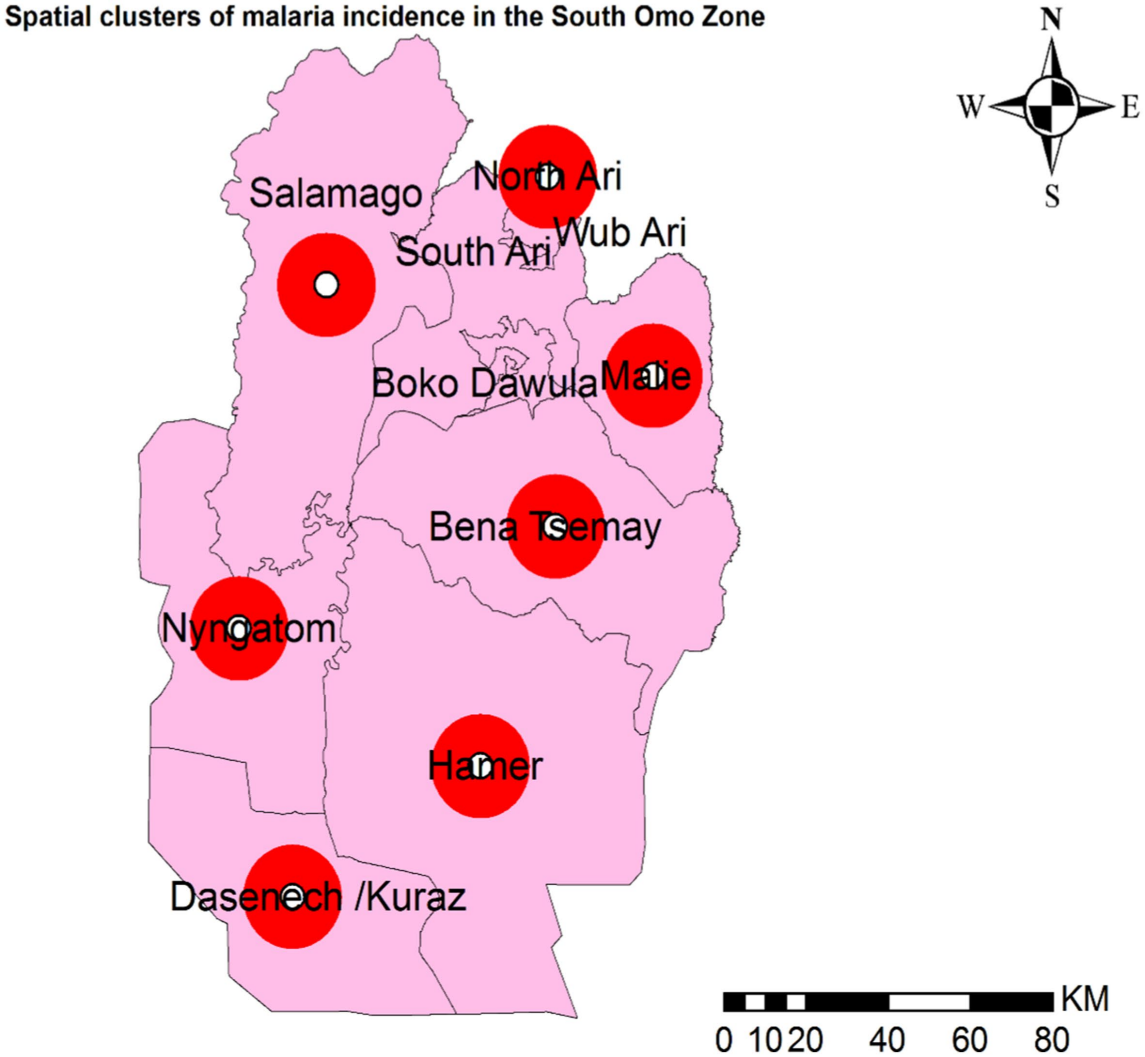
Spatial clusters of malaria incidence in the South Omo Zone, Southwest Ethiopia, 08th July 2019- 07th July 2023. The red circles in the figure represent woredas (districts) with spatial clusters of malaria incidence where the *p*-value is less than 0.0001. Source of shapefile: Ethiopian Statistical Service, own map output from ArcGIS V.10.7.

Primary spatial clusters were identified in Dasenech (RR = 2.06, *p* < 0.0001), while secondary clusters were detected in Hamer (RR = 1.90, *p* < 0.0001), Salamago (RR = 2.00, *p* < 0.0001), Bena Tsemay (RR = 1.71, *p* < 0.0001), Malie (RR = 1.50, *p* < 0.0001), Nyangatom (RR = 1.91, *p* < 0.0001), and North Ari (RR = 1.05, *p* < 0.0001) districts between 08th July, 2019, and 07th July, 2023. No clusters were identified in Jinka Town, South Ari, Wub Ari, or Boko Dawula (Table 1 and Figure 5).

**Table 1:** Spatial clusters of malaria incidence in the South Omo Zone, Southwest Ethiopia, 08th July 2019- 07th July 2023.

Cluster	Woreda/district	Population	XY coordinates	Observed number of malaria incidence	Expected number of malaria incidence	Relative ratio	LLR
1	Dasenech	79,867	(4.684659 N, 36.090778 E)/0 km	205,846	109536.05	2.06	37995.52
2	Hamer	83,803	(4.955257 N, 36.502528 E)/0 km	200,608	114934.09	1.90	29584.85
3	Salamago	42,217	(5.951219 N, 36.165150 E)/0 km	110,235	57900.00	2.00	19878.06
4	Bena Tsemay	80,156	(5.451280 N, 36.666493 E)/0 km	176,260	109931.29	1.71	18971.83
5	Male	126,941	(5.763219 N, 36.880938 E)/0 km	242,765	174096.70	1.50	14431.17
6	Nyngatom	26,348	(5.240837 N, 35.973667 E)/0 km	67,166	36135.06	1.91	11027.9
7	North Ari	95,200	(6.175410 N, 36.649943 E)/0 km	136,606	130564.84	1.05	154.97

LLR, log-likelihood ratio; RR, relative ratio.

### 3.5 Temporal cluster

Temporal malaria clusters were observed across all districts from 08th July 2022 to 07th July (RR = 1.59, *p* = 0.001) (Table 2).

**Table 2:** Temporal clusters of malaria incidence in the South Omo Zone, Southwest Ethiopia, from 08th July 2019 to 07th July 2023.

Cluster	Woreda/district	Period	Observed number of malaria incidence	Expected number of malaria incidence	Relative ratio (RR)	LLR	*p*-value
1	All	08th July 2022 to 07th July 2023	420,847	304241.45	1.59	28010.61	0.001

LLR, log-likelihood ratio.

### 3.6 Spatiotemporal clusters

Spatiotemporal malaria clusters were detected in eight districts, namely Jinka town from 2022/08/07 to 2023/07/07 (RR = 1.19, *p* < 0.001), North Ari from 2021/08/07 to 2022/07/07 (RR = 1.50, *p* < 0.001), Nyngatom from 2022/08/07 to 2023/07/07 (RR = 2.65, *p* < 0.001), Bena Tsemay from 2021/08/07 to 2022/07/07 (RR = 1.80, *p* < 0.001), Malie from 2022/08/07 to 2023/07/07 (RR = 2.03, *p* < 0.001), Hamer from 2021/08/07 to 2022/07/07 (RR = 1.95, *p* < 0.001), Salamago from 2022/08/07 to 2023/07/07 (RR = 2.97, *p* < 0.001), and Dasenech from 2022/08/07 to 2023/07/07 (RR = 2.26, *p* < 0.001), hierarchically from least to most clusters (Table 3).

**Table 3:** Spatiotemporal clusters of malaria incidence in the South Omo Zone, Southwest Ethiopia, 08th July 2019 to 07th July 2023.

Cluster	Woreda/district	Period	Observed number of malaria incidence	Expected number of malaria incidences	Relative ratio (RR)	LLR	*p*-value
1	Dasenech	2021/08/07 to 2022/07/07	120,082	56220.12	2.26	29108.11	<0.001
2	Salamago	2022/08/07 to 2023/07/07	43,660	15049.95	2.97	18242.73	<0.001
3	Hamer	2021/08/07 to 2022/07/07	20,725	55088.03	1.95	17337.01	<0.001
4	Malie	2022/08/07 to 2023/07/07	88,244	45201.63	2.03	16812.77	<0.001
5	Bena Tsemay	2021/08/07 to 2022/07/07	97,966	56417.63	1.80	13286.65	<0.001
6	Nyngatom	2022/08/07 to 2023/07/07	24,491	9378.51	2.65	8493.50	<0.001
7	North Ari	2021/08/07 to 2022/07/07	92,049	63123.46	1.50	6173.74	<0.001
8	Jinka town	2022/08/07 to 2023/07/07	22,982	19319.15	1.19	332.91	<0.001

LLR, log-likelihood ratio.

## 4 Discussion

This retrospective study revealed spatial, temporal, and spatiotemporal clusters of malaria incidence in the South Omo Zone. The rationale for conducting spatial, temporal, and spatiotemporal analyses of malaria incidence separately lies in each analysis’s unique insights, collectively offering a comprehensive understanding of malaria patterns. Spatial analysis identifies high-risk regions or hotspots, guiding targeted interventions, but lacks information on temporal changes. Temporal analysis detects trends, seasonality, or heightened transmission periods, which are essential for timing interventions but treat all regions as a single unit. The spatiotemporal analysis integrates both dimensions (spatial and temporal), providing a dynamic view of disease spread over time and space, though it requires complex models and may obscure specific insights. Using all three approaches uncovers complementary aspects—spatial analysis reveals static hotspots, temporal analysis identifies timing, and spatiotemporal analysis captures evolving patterns—ensuring robust, actionable findings for effective resource allocation and interventions for malaria.

Between 2019 and 2023, spatial malaria clusters were detected, with primary clusters identified in Dasenech and secondary clusters in North Ari, Nyangatom, Malie, Bena Tsemay, Salamago, and Hamer. Our findings are consistent with studies conducted in northwest Ethiopia and at the village level in areas with unstable malaria transmission in Ethiopia, which similarly identified spatial variations across their respective study areas (38, 39). These can be due to several environmental factors in these woredas, such as temperature and rainfall. Since most of these woredas are lowlands (40, 41), the temperature makes it easier for mosquitos to reproduce and survive (42). In addition, rainfall creates stagnant water bodies, which are ideal breeding grounds for mosquitos (43). The majority of these districts are lowlands with poor drainage and heavy rainfall, resulting in additional breeding areas (43, 44). Furthermore, high humidity levels, which are common in many lowland environments, promote mosquito breeding and survival in this district (44). Finally, the population in this district (Dasenech, Hamer, Salamago, Bena Tsemay, Malie, Nyngatom, and North Ari) has higher poverty rates, which makes people more vulnerable to malaria because they have limited access to preventive measures such as bed nets and insecticides, as well as proper healthcare (45).

This study showed there were temporal malaria clusters between 08th July 2022 and 07th July 2023. This can be due to disruptions in the economy, social infrastructures, health facilities, and services caused by the Coronavirus Disease 2019 (COVID-19) pandemic. The sudden surge in malaria cases in 2022/23 may be attributed to ongoing armed conflicts and unrest, which disrupted health services and interventions (46–48). Disruptions caused by the COVID-19 pandemic and ongoing conflicts have severely impacted malaria prevention and treatment (49). Overburdened health systems have deprioritized control measures such as insecticide-treated nets (ITNs) and indoor residual spraying (IRS), while conflicts damage infrastructure and disrupt antimalarial supply chains (49). Displaced populations face overcrowded, unsanitary conditions with inadequate mosquito control, increasing malaria risk (50). Finally, significant spatiotemporal malaria clusters were detected in Jinka town from 2022/08/07 to 2023/07/07, North Ari from 2021/08/07 to 2022/07/07, Nyngatom from 2022/08/07 to 2023/07/07, Bena Tsemay from 2021/08/07 to 2022/07/07, Malie from 2022/08/07 to 2023/07/07, Hamer from 2021/08/07 to 2022/07/07, Salamago from 2022/08/07 to 2023/07/07, and Dasenech from 2022/08/07 to 2023/07/07, hierarchically from least to most clusters. The primary reason for this phenomenon could be that pastoralist groups in the South Omo Zone are susceptible to the introduction of malaria parasites into new areas and the facilitation of disease transmission between communities due to their distinctive patterns of human movements, such as seasonal migrations in search of water and grazing pasture for cattle. Furthermore, poor infrastructure, rural locations, cultural barriers, and limited access to healthcare services can all contribute to limited access to healthcare services in pastoralist areas, which can delay diagnosis and treatment and raise the incidence of malaria. These findings are supported by a study conducted in Northeast Ethiopia, Southern Ethiopia, and Senegal (51–53).

The incidence of malaria exhibited interannual variability across districts and survey years. This finding is supported by a study conducted in Mozambique (54), Senegal (55), Zimbabwe (56), Burkina Faso (57), West Gojjam (58), and Northwest Ethiopia (59, 60). They revealed spatiotemporal variation of malaria incidence.

This variation could be due to geographical and meteorological differences, WASH infrastructure conditions, and socioeconomic and environmental factors.

## 5 Conclusion

This study found spatial, temporal, and spatiotemporal clusters in malaria incidence in the South Omo Zone. Further research is needed to investigate the factors driving the elevated malaria risk in the identified clusters. This should include an examination of individual, household, geographical, and climatic characteristics to gain a more thorough understanding of malaria risk.

### 5.1 Strength of the study

This study is the first attempt in the region. This study will provide a baseline for evaluating the progression of malaria elimination programs, as the spatial, temporal, and spatiotemporal analysis enables tracking changes in malaria incidence over time and across the study area, assessing the effectiveness of strategies such as case management, vector control, and surveillance efforts. Moreover, it is methodologically solid and thoroughly explains the spatial, temporal, and spatiotemporal clusters in malaria incidence across populations. It also serves as a starting point for further investigation into what distinguishes hot spot districts from cold spot districts, allowing us to focus on these risk factors.

### 5.2 Limitations of the study

The data were gathered using a passive surveillance system, meaning that individuals may not report to formal governmental health institutions when they fall ill. Instead, they choose traditional medicine or purchase drugs from pharmacies while they are at home. Moreover, biases can occur with Rapid Diagnostic Tests (RDTs) for malaria due to factors such as user-related biases, where misinterpretation by untrained healthcare providers can lead to overdiagnosis, especially in areas with high malaria prevalence. RDTs might be more accurate in some regions of the study area due to better healthcare infrastructure or availability of resources. In contrast, limited access to quality testing or treatment options in underserved areas could lead to biased results.

Additionally, the data collected during the COVID-19 pandemic may have introduced bias. Furthermore, this study should include host (individual) variables and household, socioeconomic, environmental, and organizational aspects. Unfortunately, data for these factors were not available.

## Data Availability

The raw data supporting the conclusions of this article will be made available by the authors without undue reservation.
